# Senolytic Treatment With Fisetin Reverses Age‐Related Endothelial Dysfunction Partially Mediated by SASP Factor CXCL12

**DOI:** 10.1111/acel.70500

**Published:** 2026-04-22

**Authors:** Sophia A. Mahoney, Krystyna Mazan‐Mamczarz, Dimitrios Tsitsipatis, Nicholas S. VanDongen, Charnae’ Henry‐Smith, Ada N. Okereke, Rachel Munk, Sanna Darvish, Kevin O. Murray, Supriyo De, Myriam Gorospe, Douglas R. Seals, Matthew J. Rossman, Allison B. Herman, Zachary S. Clayton

**Affiliations:** ^1^ Department of Integrative Physiology University of Colorado Boulder Boulder Colorado USA; ^2^ Laboratory of Genetics and Genomics National Institute on Aging Intramural Research Program, National Institutes of Health Baltimore Maryland USA; ^3^ Laboratory of Cardiovascular Science National Institute on Aging Intramural Research Program, National Institutes of Health Baltimore Maryland USA; ^4^ Department of Medicine–Geriatrics University of Colorado Anschutz Medical Campus Aurora Colorado USA

**Keywords:** cellular senescence, CXCL12, endothelial dysfunction, senolytics, vascular aging

## Abstract

Advancing age is the strongest risk factor for cardiovascular diseases (CVDs), primarily due to progressive vascular endothelial dysfunction. Cellular senescence and the senescence‐associated secretory phenotype (SASP) contribute to age‐related endothelial dysfunction by promoting mitochondrial oxidative stress and inflammation, which reduce nitric oxide (NO) bioavailability. However, the molecular changes in senescent endothelial cells (ECs) and their role in endothelial dysfunction with aging remain unclear. As such, we sought to identify the EC‐related signaling pathways, endothelial‐associated SASP factors, and their impact on endothelial function with aging. Single‐cell transcriptomics was performed on aortas from young (6mos) and old (27mos) female and male mice with and without in vivo senolytic treatment with fisetin (100 mg/kg/day administered intermittently) to characterize EC senescence and transcript expression changes. Circulating levels of SASP factors were measured to assess systemic changes associated with aging and fisetin treatment. Plasma exposure experiments were conducted in isolated mouse arteries and cultured human aortic ECs to determine the causal role of the circulating SASP milieu and specific SASP factors in mediating endothelial dysfunction and underlying mechanisms of action. Senescent ECs exhibited elevated expression of SASP factors, particularly *Cxcl12*, which was reversed by fisetin supplementation, with responses also reflected in circulating CXCL12 concentrations. Plasma from old mice impaired endothelial function by inducing vascular cell senescence, reducing NO, increasing mitochondrial oxidative stress, shifting receptor and promoting endothelial‐to‐mesenchymal transition—effects partially driven by CXCL12 and prevented by fisetin. These results identify the SASP and CXCL12 as drivers of age‐related endothelial dysfunction and establish mechanisms of senolytic intervention with fisetin supplementation.

## Graphical abstract

Single‐cell transcriptomics identifies that oral, intermittent fisetin supplementation improves endothelial function by targeting senescent endothelial cell‐derived senescence‐associated secretory phenotype (SASP) factors and CXCL12 in old animals. The SASP and specifically CXCL12 were found to drive age‐related endothelial dysfunction by promoting vascular cell senescence, stimulating mitochondrial oxidative stress, reducing nitric oxide bioavailability, and supporting endothelial cell transdifferentiation.

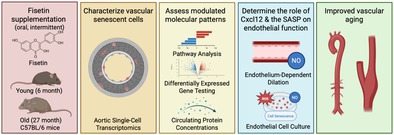

## 1 Introduction

Advancing age is the primary risk factor for cardiovascular diseases (CVDs), which remain the leading cause of morbidity and mortality worldwide (Olshansky et al. 2009; Martin et al. 2024). Impaired vascular endothelial function is a key antecedent to the development of clinical CVDs with aging (Lakatta and Levy 2003). The hallmark of aging, cellular senescence, directly contributes to age‐related endothelial dysfunction, in part by promoting chronic inflammation and mitochondrial oxidative stress (López‐Otín et al. 2023; Campisi 2013; Clayton et al. 2023; Campisi and d'Adda di Fagagna 2007). Despite the adverse effects of cellular senescence on endothelial function, the molecular changes that occur in senescent endothelial cells (ECs) and the direct mechanisms by which senescent cells induce endothelial dysfunction with advanced age remain incompletely defined.

A putative mechanism by which senescent cells drive physiological dysfunction is through the production and secretion of the circulating senescence‐associated secretory phenotype (SASP) milieu, a collection of cytokines, chemokines, proteases, and growth factors (Coppé et al. 2010). The circulating SASP milieu is a highly heterogeneous and dependent on cell type and cellular senescence inducer. Thus, the specific SASP factors that are upregulated with aging in senescent ECs in vivo remain to be determined. The circulating SASP milieu may induce cellular and tissue‐level dysfunction by driving mitochondrial oxidative stress, chronic inflammation, induction of cellular senescence in neighboring cells/tissues, and cell transdifferentiation (i.e., endothelial‐to‐mesenchymal transition [EndoMT]) (Coppé et al. 2010). Importantly, the circulating SASP milieu is in constant and direct contact with the endothelium, and changes that occur in the circulation contribute directly to age‐related endothelial dysfunction (Bloom et al. 2023; Mahoney et al. 2024). As such, ECs may be more susceptible to entering a senescent state given the constant interaction with the circulating SASP milieu (Bloom et al. 2023; Yousefzadeh et al. 2020). However, the circulating SASP factors originating from senescent ECs and their potential impact on endothelial function remain incompletely understood. As such, gaining an EC‐specific understanding of the cellular senescence‐ and circulating SASP milieu‐related molecular mechanisms within the endothelium may be exploited as translational diagnostic biomarkers, and may serve as putative therapeutic targets for novel interventions.

Senolytics—compounds that selectively target and eliminate excess senescent cells (Kirkland and Tchkonia 2020; Zhu et al. 2015)—have demonstrated efficacy for suppressing excess vascular cell senescence and improving vascular function in old mice (Clayton et al. 2023; Mahoney, Venkatasubramanian, et al. 2023; Roos et al. 2016). Although several senolytic therapies are currently being advanced in preclinical and clinical studies, the natural senolytic fisetin shows efficacy in targeting senescent ECs in culture (Zhu et al. 2017) and evoking favorable effects on endothelial function with aging (Mahoney, Venkatasubramanian, et al. 2023), and has an encouraging safety profile for clinical translation (Yousefzadeh et al. 2018). However, the effects of fisetin on senescent ECs and the SASP in vivo, and how these effects contribute to the beneficial effects of fisetin on age‐related endothelial dysfunction are incompletely understood.

In the present study, we sought to characterize the vasculature from young (6 months) and old (27 months) mice, leveraging in vivo fisetin supplementation to explore the molecular, cellular, and physiological mechanisms modulated with aging and senolytic treatment. The central goals of this study were to: (1) assess how aging affects EC senescence in vivo at a single‐cell level and identify the key cellular senescence‐associated signaling pathways; (2) identify SASP factors upregulated in senescent ECs with aging that may modulate endothelial function; and (3) evaluate the contributions of the circulating SASP milieu and specific SASP factors in mediating age‐related endothelial dysfunction.

We first uncovered that in relation to other vascular cell types, ECs were highly susceptible to becoming senescent with aging and were effectively eliminated by senolytic treatment with fisetin. Within senescent ECs, we identified *Cxcl12* mRNA to be the most highly upregulated with aging and ameliorated with fisetin senolytic treatment, and we observed similar patterns in circulating concentrations of CXCL12 protein. Finally, we revealed a causal role of the circulating SASP milieu in inducing age‐related endothelial dysfunction mediated, in part, by the elevated concentration of CXCL12. Overall, these findings distinguish senescent ECs as senolytic targets, establish the circulating SASP milieu as a driver of age‐related endothelial dysfunction, and identify CXCL12 as a specific circulating SASP factor that mediates EC senescence and dysfunction with aging that can be modulated with fisetin senolytic treatment. Further, we uncover mechanisms underlying the circulating SASP milieu‐ and CXCL12‐mediated endothelial dysfunction including NO production, mitochondrial oxidative stress, and EC trandifferentiation.

## 2 Methods

### 2.1 Animals

Mouse experiments, including the import, housing, experimental procedures, and euthanasia, were performed strictly under an Animal Study Proposal (ASP #496‐LCS‐2026) and associated amendments, reviewed and approved by the Animal Care and Use Committee (ACUC) of the National Institute on Aging (NIA). All mice were group‐housed at 22°C set point ±3 under a standard 12 h light/dark cycle and fed ad libitum. Relative humidity was maintained at 30%–70%. Procedures associated with plasma‐mediated vascular endothelial function experiments were performed and approved by the University of Colorado Boulder Institutional ACUC (procotol 2618).

**FIGURE 1:**
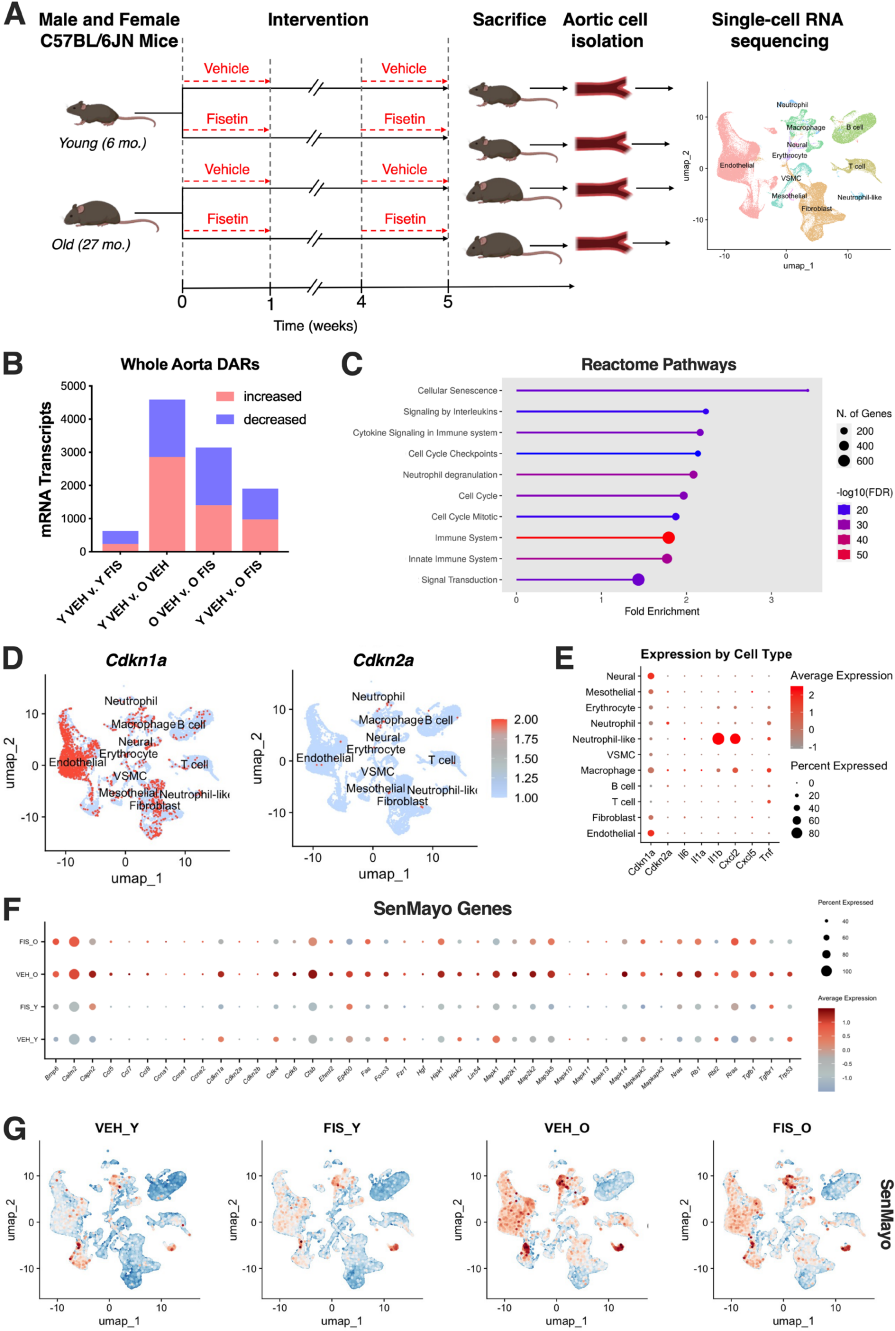
Whole aortic single‐cell RNA sequencing identifies senescent cell populations with aging that are ameliorated by senolytic treatment with fisetin. Schematic of study design used in animal model resulting in 4 groups: Young vehicle (Y VEH), young fisetin (Y FIS), old vehicle (O VEH), and old fisetin (O FIS) treated mice with combined Uniform Manifold Approximation and Projection (UMAP) (A). Number of differentially abundant RNA (DAR) between select treatment groups (B). Reactome pathway analysis of increased whole‐aorta DARs with age (O VEH vs. Y VEH) (C). Combined UMAP of canonical cellular senescence markers (D). Dot plot of canonical cellular senescence and senescence‐associated secretory phenotype (SASP) transcripts by cell type (E). Dot plot of cellular senescence‐related DARs (F). UMAP of module gene scoring using the SenMayo signature to identify senescent cells (G). Statistical analyses were conducted using Seurat's non‐parametric Wilcoxon rank sum test.

For the intervention period, young (6 months) and old (27 months) male and female C57BL/6N mice were randomly assigned to receive vehicle (10% ethanol, 30% PEG400 and 60% Phosal 50 PG) or fisetin (100 mg/kg/day in vehicle). Treatment was administered via oral gavage using an intermittent dosing paradigm—1 week on; 2 weeks off; 1 week on, as previously established senolytic dosing paradigm (Figure 1A) (Mahoney, Venkatasubramanian, et al. 2023; Yousefzadeh et al. 2018).

### 2.2 Aortic Single‐Cell RNA Sequencing

Aortic single‐cell RNA sequencing (RNAseq) analysis was performed, as previously described (Mazan‐Mamczarz et al. 2023). Briefly, whole aortas from 2‐3 mice (per sex and treatment group) were collected, cleaned and digested. Viable cells were sorted using Fluorescence‐Activated Cell Sorting for 10× Genomics library preparation. The single‐cell libraries were prepared with Chromium Next GEM Single Cell 3′ Kit v3.1 (10× Genomics) with Chromium Next GEM Chip G Single Cell Kit (10× Genomics) according to the manufacturer's protocol with Chromium Controller (10× Genomics). ~10,000 single cells were used for GEM generation, and the cDNAs were then checked on the Agilent Bioanalyzer with High Sensitivity DNA kit (Agilent). Prepared cDNAs were then used for library preparation, that were checked on the Agilent Bioanalyzer with the DNA 1000 kit (Agilent Technologies). The libraries were sequenced with an Illumina NovaSeq 6000 sequencer at a depth of 70,000–120,000 reads per cell. Sequencing data are deposited in the NCBI's Gene Expression Omnibus repository GSE296698.

Single‐cell RNAseq data were processed using Cell Ranger (version 8.0.1) with the mouse reference GRCm39‐2024‐A (10× Genomics). The obtained read count matrices were subsequently analyzed in R using the Seurat package, version 5.2.0, with default parameters in all functions, unless otherwise specified (Hao et al. 2024). Differentially abundant RNA (DAR) testing was performed with the following cutoffs: adjusted *p*‐value < 0.05, average log2 fold change > 0.25, and minimum percentage of cells in either group > 0.1 (Mazan‐Mamczarz et al. 2023). Pathway analysis was performed using KEGG (version 111.0) (Kanehisa et al. 2025) and Reactome (version 90) (Milacic et al. 2024) and was based on DAR testing. Inference and analysis of cell–cell communication was performed using CellChat (version 2.1.2) (Jin et al. 2021).

### 2.3 Plasma Protein Quantification

For multiplex analysis, custom murine Luminex Assay kits were designed by R&D Biosystems and executed according to the manufacturer's instructions.

### 2.4 Plasma‐Mediated Vascular Endothelial Function

To assess the role of the circulating SASP milieu (plasma) and CXCL12 on endothelial function, an ex vivo isolated artery bioassay was leveraged, as previously described (Mahoney et al. 2024). In brief, carotid arteries were excised from young (3–6 months), intervention‐naïve wildtype mice and cannulated onto pressure myographs. Plasma collected from young vehicle, old vehicle, and old fisetin‐treated mice was diluted to 5% and perfused luminally through the pressurized arteries for 24 h prior to assessing endothelial function. Following plasma perfusion, endothelial function was measured by endothelium‐dependent dilation (EDD) and endothelium‐independent dilation (EID) in response to increasing doses of acetylcholine (ACh) and sodium nitroprusside (SNP), respectively, as described previously (Mahoney et al. 2024; Mahoney, Venkatasubramanian, et al. 2023). All dose–response data are presented as percent dilation relative to maximum diameter to account for differences in baseline vessel diameter.

### 2.5 Cultured ECs

Human aortic ECs (HAECs) were grown in basal media supplemented with 5% plasma collected from young, old vehicle, and old fisetin‐treated mice. EC nitric oxide (NO) and mitochondrial superoxide bioactivity were assessed after 2 h plasma incubation by staining with the fluorescent probe Hoechst (nuclei stain; Thermo Fisher) and either 10 μM diaminorhodamine‐4M AM (DAR‐4M AM; Sigma‐Aldrich; to quantify NO production) for 45 min or 5 μM MitoSOX (Thermo Fisher; to quantify mitochondrial superoxide bioactivity) for 30 min. Senescence‐associated β‐galactosidase (SA‐β‐Gal) staining and RNA extractions for gene expression were performed after 24 h of plasma exposure.

### 2.6 CXCL12 Protein Addition and Inhibition

Recombinant mouse CXCL12 protein (R&D Systems, Cat. #460‐SD‐050/CF) in sterile PBS was added back to plasma from young and old fisetin‐treated mice, such that the concentration of CXCL12 matched the average levels in old vehicle‐treated mice (3210 pg/mL) for CXCL12 add‐back experiments. 1 μg/mL of LIT‐927 (Selleckchem, Cat. #S8813) (Xiong et al. 2022) in DMSO was added to the old vehicle plasma for CXCL12 inhibition experiments.

## 3 Results

### 3.1 Whole Aortic Single‐Cell Transcriptomics Identifies Senescent Cell Population With Aging That Are Ameliorated With Senolytic Treatment

To gain insights into the vascular transcriptional changes that occur with aging, we leveraged single‐cell RNAseq to compare aortas from young (6 months) and old (27 months) C57BL/6N mice. To study the effects of in vivo senolytic treatment on the vasculature, we supplemented young and old mice with vehicle or fisetin, as previously described (Mahoney, Venkatasubramanian, et al. 2023) (Figure 1A). Single‐cell RNAseq workflows allow for the characterization of transcriptomic profiles from distinct cell types of the aorta to identify cell type‐specific changes with aging (Mahoney, Dey, et al. 2023). Unsupervised clustering of the merged samples revealed 11 distinct cell types in the aorta (Figure 1A, Figure S1).

We next performed whole‐aorta pseudo‐bulk RNAseq to determine the general transcriptomic changes with aging and senolytic treatment using differentially abundant RNA (DAR) analysis. Age‐related differences (young vs. old vehicle) revealed 4589 DARs (2859 higher, 1730 lower) (Figure 1B). Fisetin supplementation in old mice unveiled 3143 DARs with more reduced transcripts than elevated ones (1401 higher, 1742 lower) compared to old vehicle (Figure 1B). Old fisetin mice had a more similar transcriptomic profile to young vehicle mice (974 higher, 931 lower) compared to old vehicle mice (Figure 1B). The young groups (young vehicle vs. young fisetin) were the least different with 628 DARs (240 higher, 388 lower) (Figure 1B). Reactome pathway analysis revealed cellular senescence as the most highly upregulated pathway with aging, followed by pathways related to immune dysregulation and cell cycle arrest (Figure 1C).

Given the changes in cellular senescence pathways observed in the vasculature with advanced age, we next sought to assess how ECs were affected relative to other vascular cell types. First, we assessed canonical cellular senescence transcripts *Cdkn1a* and *Cdkn2a* and key SASP factor transcripts to identify upregulations in endothelial, macrophage, neutrophil‐like, and neural cells (Figure 1D,E). To gain insight into the cell senescence‐related transcripts in the vasculature that were modulated with aging and senolytic treatment, we assessed genes in the holistic senescent cell signature SenMayo (Saul et al. 2022) that were upregulated with aging (old vehicle vs. young vehicle) and downregulated with senolytic treatment (old fisetin vs. old vehicle). We found differences in transcripts involved in the SASP, cell cycle arrest maintenance, and pro‐inflammatory signaling (Figure 1F). Next, we leveraged module scoring of SenMayo; we identified senescent cell populations in several vascular cell types, including endothelial, macrophage, and neutrophil‐like cell populations in aortas from old vehicle mice (Figure 1G). In the aortas from old mice, fisetin had the greatest effects on the SenMayo signature in the EC population, reinforcing the senolytic effects of fisetin on senescent EC burden with aging (Figure 1G). Together, these data demonstrate a transcriptional shift in the vasculature with aging and suggest that senolytic treatment with fisetin in old mice returns the transcriptome towards a young aortic cell composition by mitigating vascular cell senescence burden, particularly in the endothelium.

### 3.2 Senescent EC Burden Increases in the Aorta With Aging and Is Ameliorated by Senolytic Treatment

**FIGURE 2:**
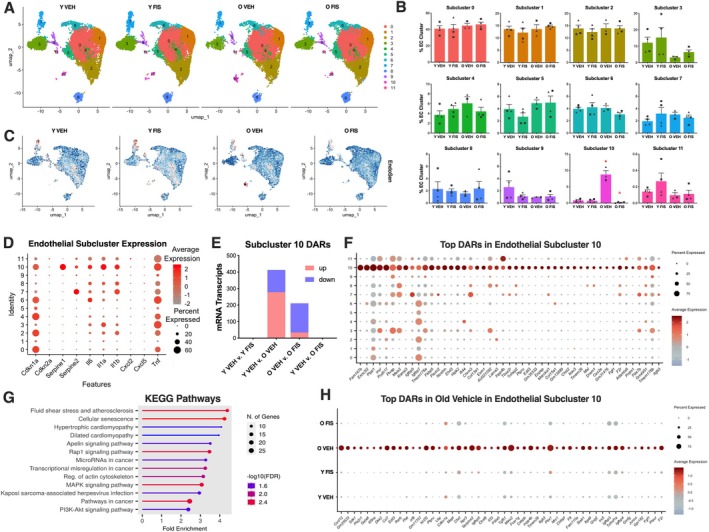
Senescent endothelial cell (ECs) increase in aortas with aging and are ameliorated by senolytic treatment with fisetin. Uniform manifold approximation and projection (UMAP) (A) and subcluster percentage (B) of aortic ECs from the young vehicle (Y VEH), young fisetin (Y FIS), old vehicle (O VEH), and old fisetin (O FIS) treated mice. Module gene scoring using EndoSen signature to identify senescent ECs (C). Dot plot of the canonical cellular senescence and senescence‐associated secretory phenotype (SASP) transcripts across endothelial clusters (D). Differentially abundant RNA (DAR) between select treatment groups in senescent endothelial subcluster 10 (E). Dot plot analysis of top 50 differentially abundant RNAs (DARs) in endothelial subcluster 10 vs. other endothelial subclusters (F). KEGG pathway analysis of increased DARs in senescent endothelial subcluster 10 vs. other endothelial subclusters (G). Dot plot analysis of the top 50 DARs in endothelial subcluster 10 in old vehicle vs. other treatment groups (H). *p < 0.05 vs. Y VEH, ^*p* < 0.05 vs. O VEH; triangle represents females, squares represent males. Statistical analyses were conducted using Seurat non‐parametric Wilcoxon rank sum test. In (*B*), statistical analyses were conducted using Prism one‐way ANOVA followed by multiple comparison *t*‐test.

We next performed a targeted interrogation of the cellular senescence‐related changes in the vascular EC population with aging and senolytic treatment. To isolate EC‐specific changes, we performed unsupervised clustering of the EC cluster, which produced 12 unique subclusters based on shared transcript expression patterns (Figure 2A). EC composition revealed minimal differences between treatment groups in the majority of subclusters, with the exception of subcluster 10, which increased with age (10.7‐fold, *p* < 0.0001) and reduced (0.02‐fold, *p* < 0.0001) back to young levels in old mice supplemented with fisetin (Figure 2B).

We next leveraged the EC‐specific senescent signature EndoSen (Guduric‐Fuchs et al. 2024) to determine which subclusters were enriched by cellular senescence‐associated signaling pathways. EndoSen module scoring revealed cellular senescence enrichment in endothelial subclusters 4, 7, 9, and 10, with the highest expression being in subcluster 10 (Figure 2C). Moreover, endothelial subcluster 10 had the highest mRNA expression of the canonical cellular senescence and SASP markers compared to the other subclusters (Figure 2D).

To further characterize the senescent ECs, we leveraged DAR testing on subcluster 10 to identify gene targets that are modulated with aging and senolytic treatment. Age‐related differences (old vs. young vehicle) revealed 435 DARs (278 increased, 135 reduced), suggesting a large impact of aging on senescent endothelial subcluster 10 (Figure 2E). Fisetin supplementation in old mice unveiled 211 DARs with more reduced than elevated transcripts (34 increased, 177 decreased) (Figure 2E). Notably, no transcripts were significantly modulated between old fisetin and young vehicle or between the young mouse groups, suggesting that senescent ECs from old mice that received senolytic treatment have a similar transcriptomic profile to that of ECs from young mice and that fisetin has minimal effects on young, healthy ECs (Figure 2E).

The top increased DARs that distinguished the putative senescent subcluster 10 from other EC subclusters represented transcripts involved in extracellular matrix remodeling, stress signaling, cell cycle maintenance, and the SASP (Figure 2F). Pathway analysis of the DARs from senescent endothelial subcluster 10 revealed pathways involved in cardiovascular pathology (i.e., atherosclerosis and cardiomyopathy), cellular senescence, cancer, and a general dysregulation of signaling pathways (Figure 2G). Finally, we identified the top 50 DARs that distinguished the old vehicle group from the other treatment conditions, representing transcripts that were most increased with aging and lower with fisetin supplementation in endothelial subcluster 10 (Figure 2H). We found that these transcripts largely encompass canonical senescence markers (e.g., *Cdkn1a*) and SASP factors. Together, these findings demonstrate increased expression of cellular senescence‐ and SASP‐related signaling pathways in ECs with aging that were ameliorated with senolytic treatment with fisetin.

### 3.3 Senolytic Treatment Mitigates the Endothelial‐Derived and Circulating SASP in Old Mice

**FIGURE 3:**
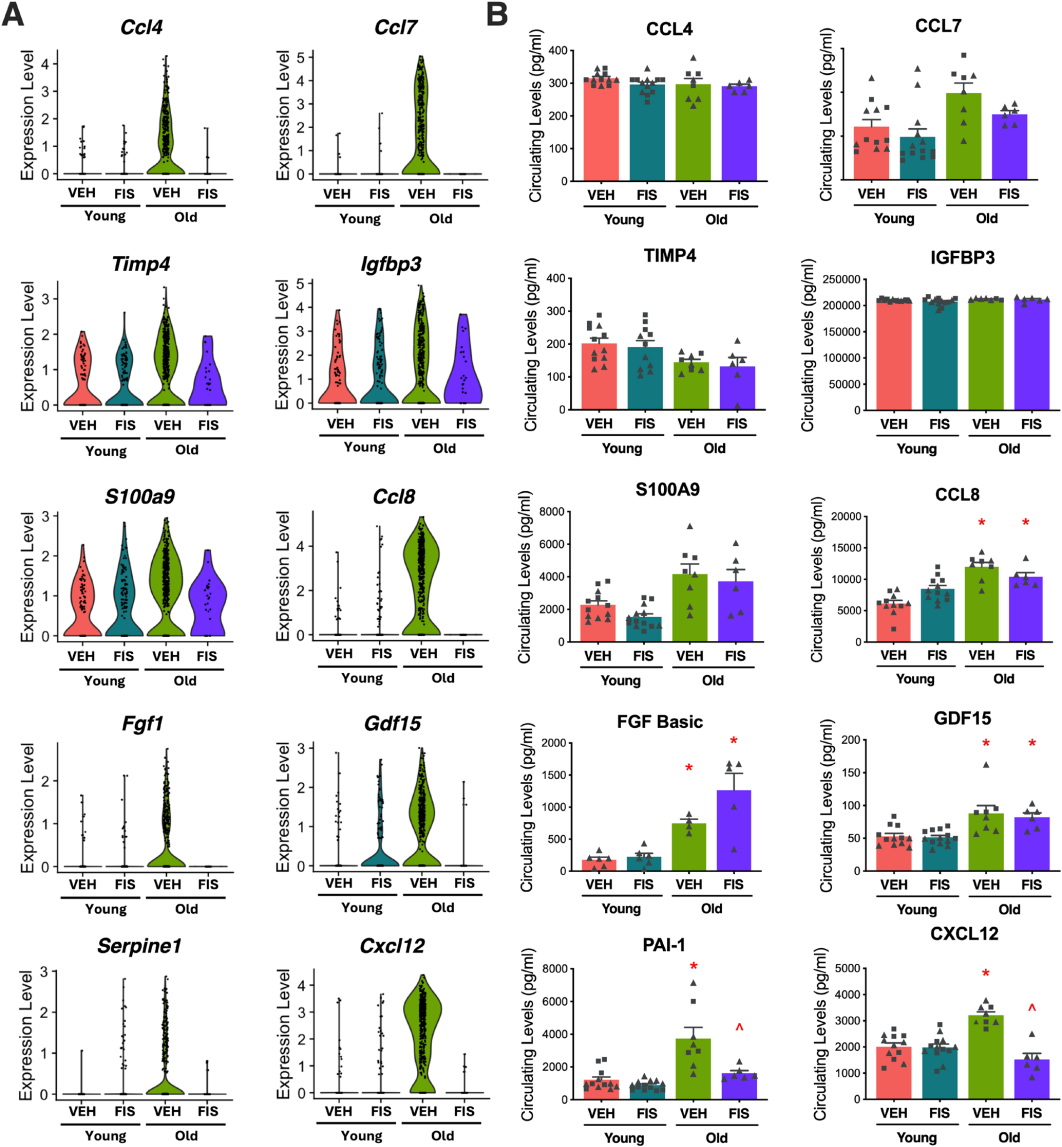
Endothelial and circulating senescence‐associated secretory phenotype (SASP) factors increase with age and are ameliorated by senolytic treatment with fisetin. Violin plots representing the top SASP‐related differentially abundant RNAs (DARs) in senescent endothelial subcluster 10 from the young vehicle (Y VEH), young fisetin (Y FIS), old vehicle (O VEH), and old fisetin (O FIS) treated mice (A). Circulating protein abundance of top SASP‐related DARs in plasma (B). *p < 0.0001 vs. Y VEH, ^*p* < 0.0001 vs. O VEH; triangle represent females, squares represent males. In (A), statistical analyses were conducted using Seurat's non‐parametric Wilcoxon rank sum test. In (*B*), statistical analyses were conducted using Prism one‐way ANOVA followed by multiple comparison *t*‐test.

The SASP represents a collection of pro‐inflammatory molecules including cytokines, chemokines, proteases, and growth factors that have varied roles in modulating physiological function (Coppé et al. 2010; Wang et al. 2024). Our next aim was to identify candidate circulating SASP factors based on those differentially expressed in the endothelium, particularly those driving the patterns observed in senescent EC subcluster 10. In EC subcluster 10, we observed several SASP factors with increased transcripts with aging (young vehicle v. old vehicle) that were reduced with fisetin (old vehicle v. old fisetin), including *Ccl4, Ccl7*, *Timp4*, *Igfbp3*, *S100a9*, *Ccl8*, *Fgf1*, *Gdf15*, *Serpine1*, and *Cxcl12* mRNAs (Figure 3A).

Provided the changes observed in mRNAs encoding SASP factors from senescent ECs, we sought to determine if these factors were translated and secreted into the circulating plasma. We selected SASP proteins to measure based on: (a) the magnitude of age‐related differences in senescent endothelial transcript expression and whether these differences with aging were influenced by fisetin supplementation; and (b) for biological plausibility of altering endothelial function. We measured the protein concentration of candidate SASP factors in the plasma from treated animals. Among these factors, higher concentrations with aging were observed in CCL8, FGF Basic, S100a9, PAI‐1, and CXCL12 when comparing young vehicle and old vehicle mice (Figure 3B). Notably, the elevated PAI‐1 and CXCL12 protein expression with aging was ameliorated by fisetin supplementation in old animals (Figure 3B).

PAI‐1, a platelet protein that inhibits fibrin degradation (Morrow et al. 2020), is a well‐established marker of cellular senescence (Vaughan et al. 2017). CXCL12 is a chemokine that regulates angiogenesis (Ara et al. 2005) and is implicated in atherosclerotic progression (Döring et al. 2019). Importantly, ECs are a key vascular source of *Cxcl12* (Figure S2A,B), and endothelial‐associated CXCL12 is a key driver of CVD^30^. Although *Cxcl12* is expressed in non‐senescent cells, it is upregulated in senescent ECs and highly correlated to canonical cellular senescence and SASP transcripts (Figure S2C–E). The enrichment of *Cxcl12* in this specific subpopulation suggests that it may serve as a localized source of SASP signaling, potentially influencing neighboring ECs through paracrine or autocrine mechanisms. Given the novelty and putative biological plausibility of CXCL12 in regulating endothelial function (whereas PAI‐1 primarily affects platelets), we next sought to investigate the targets of senescent EC‐associated CXCL12.

### 3.4 Senolytic Treatment Mitigates Senescent EC‐Derived CXCL12 Signaling

**FIGURE 4:**
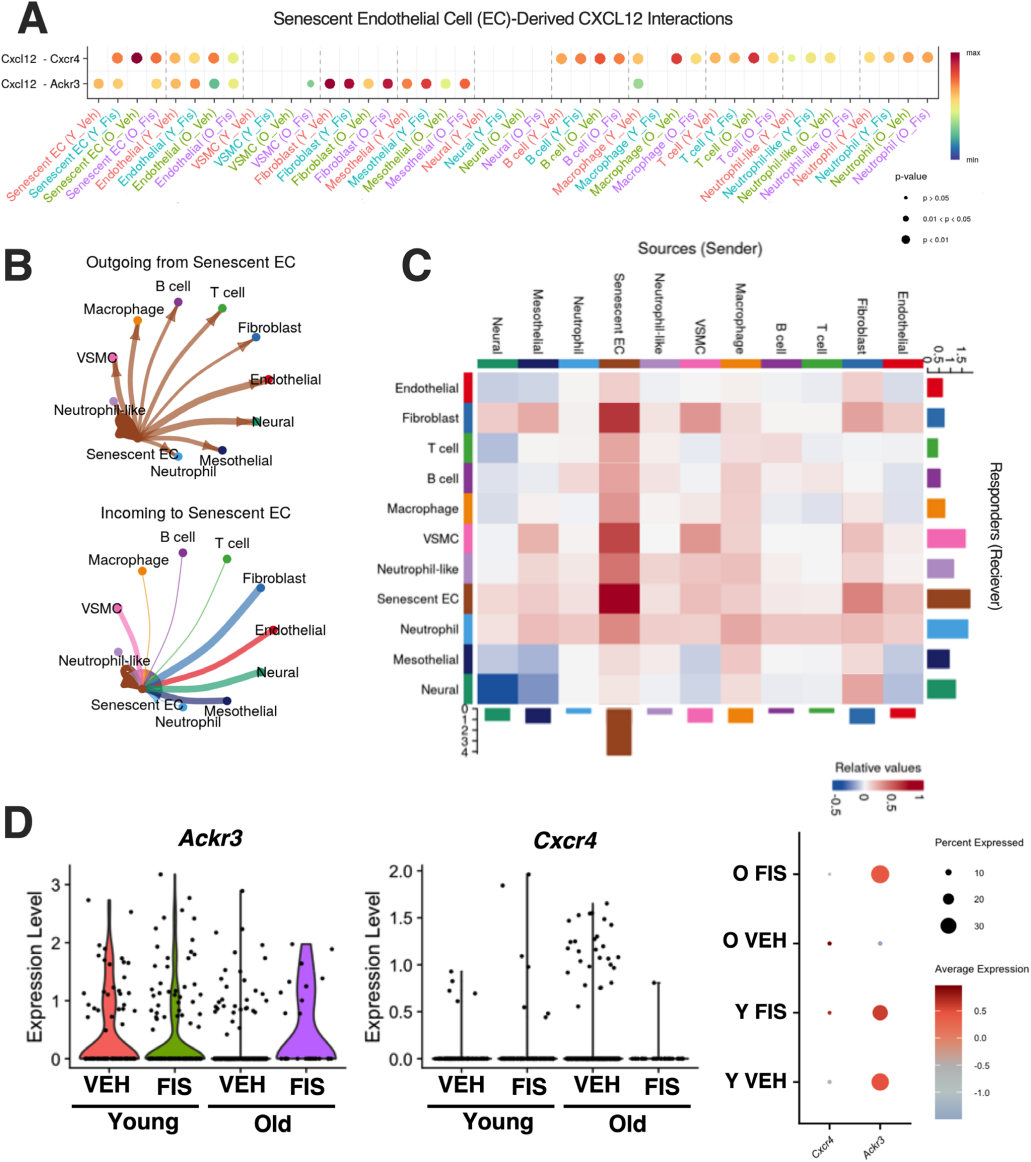
Senolytic treatment mitigates senescent endothelial cell (EC)‐derived CXCL12 signaling. Dot plot visualization of cell‐cell communication from senescent EC‐derived CXCL12 interactions across cell types from the young vehicle (Y VEH), young fisetin (Y FIS), old vehicle (O VEH), and old fisetin (O FIS) treated mice (A). Combined network diagrams of outgoing (top) and incoming (bottom) communication signals from senescent ECs (B). Combined heatmap of differential interaction strength representing the relative change in communication probability between sending and receiving cell types (C). Expression profiles of CXCL12 receptors in senescent EC cluster 10 across treatment groups (D). Statistical analyses were conducted using Seurat's non‐parametric Wilcoxon rank sum test.

To understand how senescent EC‐derived CXCL12 influences the aged vasculature, we performed an unbiased cell–cell communication analysis using the bioinformatic tool, CellChat (Jin et al. 2021), on the single‐cell RNAseq dataset. Mapping of senescent EC‐derived CXCL12 with its 2 receptors, *Ackr3* and *Cxcr4*, revealed contrasting interaction patterns (Figure 4A). *Cxcl12‐Ackr3* interactions were higher in senescent ECs from old vehicle communicating with other senescent ECs, non‐senescent ECs, B cells, macrophages, and T cells, suggesting increased autocrine signaling and immune cell signaling with aging (Figure 4A). In contrast, *Cxcl12‐Cxcr4* interactions were lower in senescent ECs from old vehicle communicating with other senescent ECs, non‐senescent ECs, fibroblast and mesothelial cells, suggesting deficient cell–cell communication among vascular cells (Figure 4A). Importantly, both these trends were reversed in old animals treated with fisetin, suggesting a return to physiological interactions.

Differential interaction strength analysis showed that the senescent ECs exhibited a robust increase in “outgoing” interaction strength compared to all other identified cell types (Figure 4B,C). These cells established extensive communication pathways targeting non‐senescent ECs, fibroblasts, and various immune populations, including macrophages and neutrophils (Figure 4B,C). Notably, we observed a strong self‐signaling signal within the senescent ECs, suggesting an autocrine signaling that may serve to reinforce the senescent phenotype (Figure 4B,C).

To determine if CXCL12 was working through this autocrine manner, we first looked at the expression of receptors *Ackr3* and *Cxcr4* in the senescent ECs. Consistently, we found that while *Ackr3* mRNA levels were lower in the old vehicle mice compared to the other groups, *Cxcr4* mRNA were higher in the old vehicle mice compared to the other groups (Figure 4D). Together, these findings suggest that senescent EC‐derived CXCL12 may act in an autocrine manner to fuel EC senescence and dysfunction. As such, we next sought to determine if CXCL12 was directly implicated in age‐related endothelial dysfunction transduced by the circulating SASP milieu and if mitigation of elevated CXCL12 was a mechanism by which fisetin supplementation elicited its benefits on endothelial function with aging.

### 3.5 Senolytic Treatment Attenuates Circulating SASP Milieu‐Induced Endothelial Dysfunction With Aging

**FIGURE 5:**
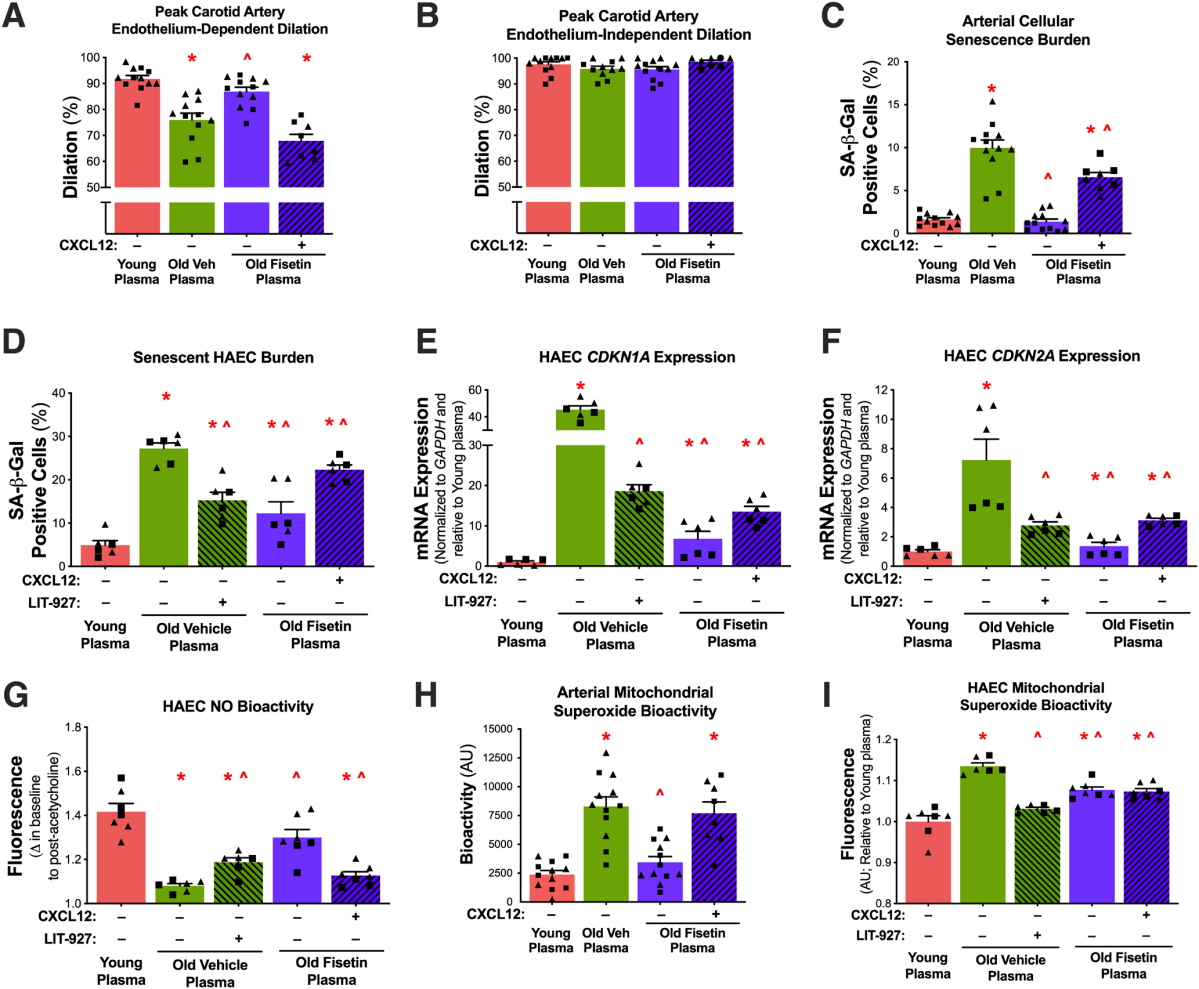
The circulating SASP‐ and CXCL12‐induced endothelial dysfunction with aging and prevention by senolytic treatment with fisetin and CXCL12 inhibition. Endothelium‐dependent dilation (A) and endothelium‐independent dilation (B) in isolated carotid arteries from young mice following plasma perfusion with and without the addition of recombinant mouse CXCL12. Senescence‐associated β‐Galactosidase (SA‐β‐Gal) signal following plasma exposure with and without the addition of recombinant CXCL12 and its inhibitor, LIT‐927 in isolated arteries (C) and human aortic endothelial cells (HAECs) (D). Cellular senescence biomarkers *CDKN1A* (E) and *CDKN2A* (F) mRNA in HAECs following plasma exposure with and without the addition of recombinant CXCL12 and LIT‐927. Nitric oxide bioactivity in HAECs following plasma exposure with and without the addition of recombinant CXCL12 and LIT‐927 (G). Mitochondrial superoxide bioactivity following plasma exposure with and without the addition of recombinant CXCL12 and LIT‐927 in isolated arteries (H) and HAECs (I). Values represent mean ± SEM; *p < 0.05 vs. young control, ^*p* < 0.05 vs. old vehicle; triangles represent females, squares represent males. Statistical analyses were conducted using Prism, one‐way ANOVA followed by multiple comparison *t*‐test.

*Circulating SASP milieu‐ and CXCL12‐induced endothelial dysfunction*. Given the observation that select circulating SASP factors changed with aging and senolytic treatment with fisetin, we next aimed to evaluate the functional role of the circulating SASP environment on age‐related endothelial dysfunction and the specific effects of CXCL12 as a candidate circulating SASP factor that might be responsible for transducing the effect of the circulating environment on endothelial function. We first tested the effects of the plasma from the young vehicle, old vehicle, and old fisetin groups on endothelial function using carotid artery endothelium‐dependent dilation (EDD) following intraluminal plasma perfusion. Average peak EDD was lower following exposure to plasma from old vehicle mice compared to young mice (−17%; *p* < 0.0001), suggesting that exposure to the aged systemic environment is sufficient to impair endothelial function (Figure 5A, Figure S3B). Peak EDD was 14% higher following exposure to plasma from old fisetin supplemented mice compared to plasma from old vehicle mice (*p* = 0.001) (Figure 5A, Figure S3A,B).

To evaluate the functional contributions of circulating CXCL12 to the impairment observed with the aged SASP milieu, we added back recombinant mouse CXCL12 to old fisetin plasma such that the average concentrations of plasma CXCL12 were similar to that of old vehicle plasma. The addition of CXCL12 to old fisetin plasma reduced peak EDD compared to old fisetin plasma alone (−22%, *p* < 0.0001), ameliorating differences with the old vehicle group (*p* = 0.465) (Figure 5A, Figure S3A,B). No differences in endothelium‐independent dilation were observed between any of the groups or conditions (*p* = 0.342), indicating that differences observed in response to plasma exposure occurred in an endothelium‐dependent manner (Figure 5B, Figure S3C,D). Together, these data suggest that the circulating SASP milieu underlies age‐related endothelial dysfunction and its detrimental effects are associated with elevated CXCL12 and largely reversed by senolytic treatment with fisetin.

*Circulating SASP‐ and CXCL12‐induced vascular cell senescence*. One mechanism by which the SASP elicits its adverse effects is through a humoral‐based induction of cellular senescence (Clayton et al. 2023; Wang et al. 2024). As such, to investigate the mechanisms by which the circulating SASP modulates endothelial function, we sought to assess SASP‐induced cellular senescence by measuring the canonical marker of senescent cell burden senescence‐associated β‐galactosidase (SA‐β‐Gal) intensity (Kurz et al. 2000; González‐Gualda et al. 2021) in arteries (aortic rings) exposed to plasma from young vehicle, old vehicle, and old fisetin mice. We found that when normalized to total cell count there was an increase in SA‐β‐Gal positive cells in arteries exposed to old versus young plasma (6.1‐fold, *p* < 0.0001) (Figure 5C, Figure S4A). SA‐β‐Gal positive cells were lower in arteries exposed to plasma from old fisetin‐treated mice compared to old vehicle mice (−86%, *p* < 0.0001) (Figure 5C, Figure S4A). The addition of exogenous CXCL12 to old fisetin plasma increased arterial SA‐β‐Gal positive cells (4.8‐fold, *p* < 0.0001) compared to old fisetin plasma alone (Figure 5D, Figure S4A).

To isolate the effects of plasma exposure on ECs, we exposed plasma from the treated animals to cultured human aortic ECs (HAECs). Similar to arteries, HAECs exposed to old vehicle plasma had greater SA‐β‐Gal intensity than HAECs exposed to young vehicle plasma (5.6‐fold, *p* < 0.0001) (Figure 5D, Figure S4B). SA‐β‐Gal positive cells were lower in HAECs exposed to plasma from old fisetin‐treated mice compared to old vehicle mice (−56%, *p* < 0.0001) (Figure 5D, Figure S4B). Inhibition of CXCL12 with LIT‐927 (a neutraligand of CXCL12 (Regenass et al. 2018)) in old vehicle plasma reduced SA‐β‐Gal positive cells (−44%, *p* < 0.0001) compared to old vehicle plasma alone (Figure 5D, Figure S4B). The addition of exogenous CXCL12 to plasma increased SA‐β‐Gal positive cells in HAECs exposed to old fisetin plasma (82%, *p* < 0.0001) compared to old fisetin plasma alone (Figure 5D, Figure S4B).

In addition to SA‐β‐Gal activity, we confirmed the effects of the SASP and CXCL12 on cellular senescence through the endothelial mRNA expression of the canonical cellular senescence biomarkers *Cdkn1a* and *Cdkn2a*. We found that HAECs exposed to plasma from old vehicle mice had higher *Cdkn1a* (45‐fold, *p* < 0.0001) and *Cdkn2a* (6.2‐fold, *p* < 0.0001) mRNA expression compared to young plasma (Figure 5E,F, Figure S4A). Relative to old vehicle plasma exposure, old fisetin plasma exposure resulted in lower *Cdkn1a* (−85%, *p* < 0.0001) and *Cdkn2a* (−57%, p < 0.0001) mRNA expression (Figure 5E,F, Figure S4C,D). The addition of LIT‐927 to old vehicle plasma reduced *Cdkn1a* (−59%, *p* < 0.0001) and *Cdkn2a* (−62%, p < 0.0001) mRNA expression compared to old vehicle plasma alone (Figure 5E,F, Figure S4C,D). The addition of CXCL12 to old fisetin plasma increased *Cdkn1a* (100%, *p* < 0.0001) and *Cdkn2a* (128%, p < 0.0001) mRNA expression compared to old fisetin plasma alone (Figure 5E,F, Figure S4C,D). In combination, these data indicate that the circulating SASP milieu directly induces vascular EC senescence, in part through CXCL12, and that circulating SASP milieu‐induced senescence with aging may be prevented by fisetin supplementation.

*Circulating SASP‐ and CXCL12‐induced NO production and mitochondrial superoxide bioactivity*. Age‐related endothelial dysfunction is primarily mediated by reduced nitric oxide (NO) bioavailability, which is a key feature of senescent ECs (Hayashi et al. 2008). Therefore, we assessed NO production in cultured ECs exposed to plasma, examining the effects of both the addition and inhibition of CXCL12. This allowed us to determine the direct role of CXCL12 in mediating differences in EC NO production induced by the circulating SASP milieu in the context of aging and fisetin supplementation. HAECs exposed to old vehicle plasma exhibited lower NO production compared to young plasma (−24%, *p* < 0.0001) (Figure 5G, Figure S5A). This age‐related impairment in NO production was prevented in HAECs exposed to old fisetin plasma (20% vs. old vehicle plasma, *p* < 0.0001) (Figure 5G, Figure S5A). Compared to plasma alone, the addition of exogenous CXCL12 impaired NO production in HAECs exposed to young vehicle (−20%, *p* < 0.0001) and old fisetin (−13%, *p* < 0.0001) plasma (Figure 5G, Figure S5A). Further, CXCL12 inhibition by LIT‐927 increased NO production in HAECs exposed to old vehicle plasma (10%, *p* < 0.0001) (Figure 5G, Figure S5A).

Reduced NO production may occur as a result of mitochondrial oxidative stress, as excessive superoxide scavenges NO to reduce its bioavailability (Erusalimsky 2009). Thus, we sought to determine whether the circulating SASP milieu increased mitochondrial superoxide bioactivity and if this was mediated by CXCL12. To do so, we next measured mitochondrial‐derived superoxide bioactivity in isolated arteries (Figure 5H, Figure S5B) and cultured HAECs (Figure 5I, Figure S5C) following plasma exposure with the addition and inhibition of CXCL12. Arterial mitochondrial superoxide bioactivity was higher in aortas exposed to old vehicle plasma compared to young plasma (3.5‐fold, *p* < 0.0001) (Figure 5H, Figure S5B). Mitochondrial superoxide bioactivity was lower in arteries exposed to plasma from old fisetin‐treated mice compared to old vehicle‐treated mice (−58%, *p* < 0.0001) (Figure 5H, Figure S5B). Compared to old fisetin plasma alone, the addition of exogenous CXCL12 increased mitochondrial superoxide bioactivity (2.2‐fold, *p* = 0.001) (Figure 5H, Figure S5B).

Similar to arteries, HAECs exposed to old vehicle plasma had greater basal mitochondrial superoxide bioactivity than young plasma (14%, *p* < 0.0001) (Figure 5I, Figure S5C). Mitochondrial superoxide bioactivity was lower in HAECs exposed to plasma from old fisetin‐treated mice compared to old vehicle mice (−6%, *p* < 0.0001) (Figure 5I, Figure S5C). CXCL12 inhibition with LIT‐927 reduced mitochondrial superoxide bioactivity in HAECs exposed to old vehicle plasma (−10%, *p* < 0.0001) (Figure 5I, Figure S5C). Exogenous CXCL12 induced mitochondrial superoxide bioactivity in HAECs exposed to old fisetin plasma (5%, *p* = 0.029) (Figure 5I, Figure S5C). Together, these data suggest that the circulating SASP milieu impairs NO production and promotes excess mitochondrial superoxide bioactivity in senescent ECs and these SASP‐mediated differences are driven in part by CXCL12 and may be prevented by fisetin supplementation.

### 3.6 Senolytic Treatment Mitigates Endothelial‐To‐Mesenchymal Transition (EndoMT) With Aging

**FIGURE 6:**
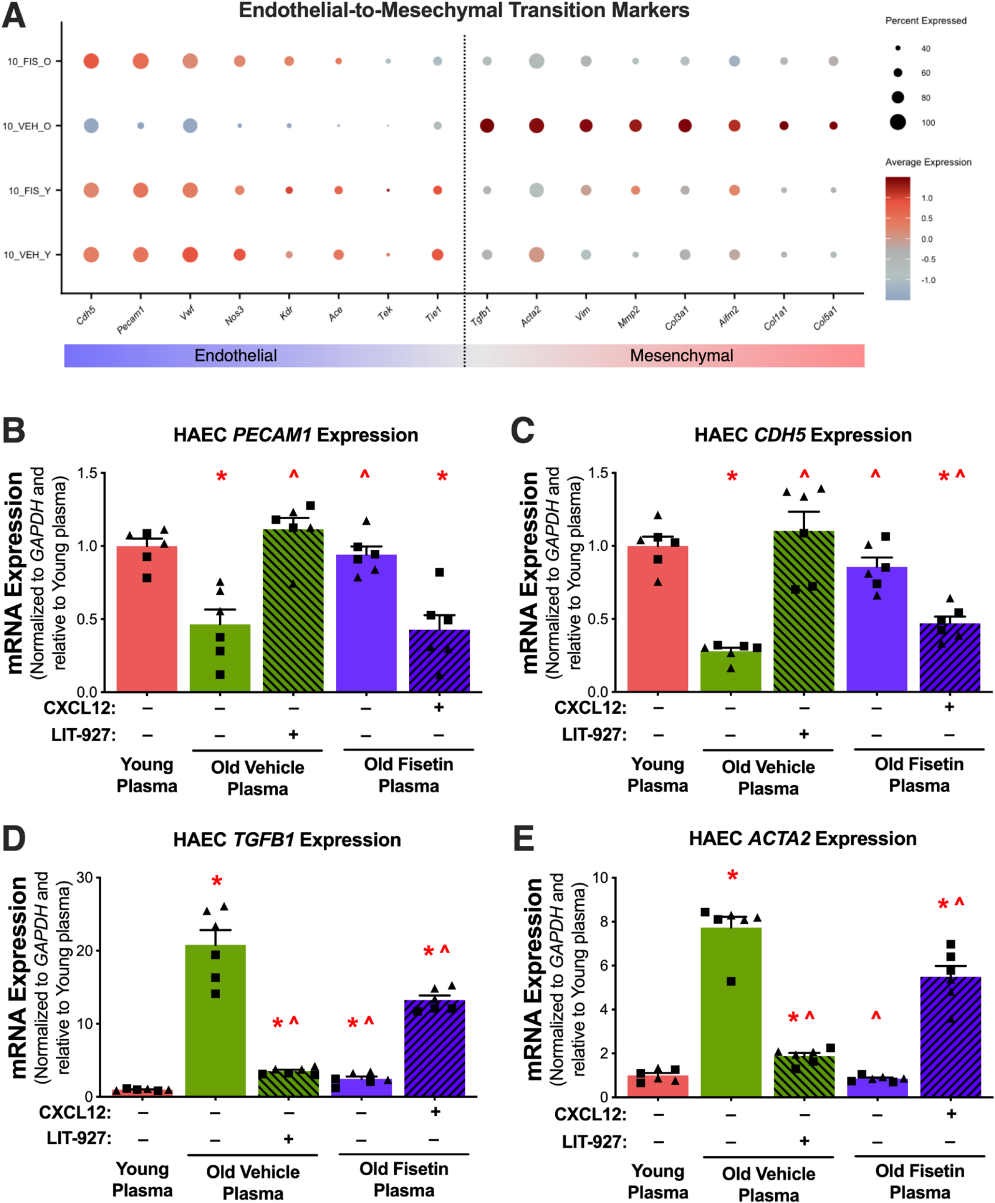
Fisetin supplementation prevents circulating SASP‐ and Cxcl12‐induced endothelial‐to‐mesenchymal transition (EndoMT). Dot plot of endothelial and mesenchymal mRNA cell markers in senescent endothelial subcluster 10 (A). mRNA levels of endothelial cell biomarkers *CDH5* (B) and *PECAM1* (C) and mesenchymal cell biomarkers *TGFB1* (D) and *ACTA2* (E) in human aortic endothelial cells (HAECs) following plasma exposure with or without the addition of recombinant CXCL12 and its inhibitor, LIT‐927. Values represent mean ± SEM; *p < 0.05 vs. young control, ^*p* < 0.05 vs. old vehicle; triangles represent females, squares represent males. In (A), statistical analyses were conducted using Seurat's non‐parametric Wilcoxon rank sum test. In (B–E), statistical analyses were conducted using Prism one‐way ANOVA followed by multiple comparison *t*‐test.

CXCL12 plays a crucial role in promoting the EndoMT, a transdifferentiation process by which ECs lose their characteristic traits while acquiring the contractile features of mesenchymal cells (Fleenor et al. 2012; Ramadhiani et al. 2021; Mahoney et al. 2025). Importantly, the EndoMT is highly implicated in vascular aging and previous studies report shifts in senescent ECs towards more contractile properties and reduced NO production (Fleenor et al. 2012; Ramadhiani et al. 2021). As such, we next sought to determine if the EndoMT was an underlying mechanism mediating SASP‐ and CXCL12‐induced dysfunction in senescent ECs. To do so, we first investigated whether senescent ECs exhibit characteristics of EndoMT and then assessed whether fisetin supplementation could prevent EndoMT induced by the circulating SASP and CXCL12. We initially measured transcripts specific to ECs and mesenchymal cells in the senescent endothelial subcluster 10 and found that the old vehicle group demonstrated reduced endothelial characteristics and higher mesenchymal markers compared to the other groups (Figure 6A).

To determine whether the age‐related circulating SASP milieu promotes EndoMT and whether these effects are mediated by CXCL12, we exposed HAECs to plasma from treated mice, either with CXCL12 added or inhibited. We then assessed the expression of the most affected endothelial markers (*Cdh5* and *Pecam1* mRNA) and mesenchymal markers (*Tgfb1* and *Acta2* mRNA). HAECs exposed to plasma from aged vehicle‐treated mice showed significantly reduced mRNA expression of *Cdh5* (−72%, *p* < 0.0001) and *Pecam1* (−54%, *p* < 0.0001) compared to cells exposed to plasma from young mice. This age‐related increase was mitigated by exposure to old fisetin plasma in *Cdh5* (3.1‐fold, *p* < 0.0001) and *Pecam1* (2‐fold, *p* < 0.0001) mRNA expression compared to old vehicle plasma (Figure 6B,C, Figure S6A,B). The addition of LIT‐927 to old vehicle plasma increased *Cdh5* (4‐fold, *p* < 0.0001) and *Pecam1* (2.4‐fold, p < 0.0001) mRNA expression compared to old vehicle plasma alone (Figure 6B,C, Figure S6A,B). The addition of CXCL12 to old fisetin plasma reduced *Cdh5* (−45%, *p* < 0.0001) and *Pecam1* (−55%, *p* < 0.0001) mRNA expression compared to old fisetin plasma alone (Figure 6B,C, Figure S6A,B).

Contrary to the observed changes in endothelial markers, HAECs exposed to plasma from old vehicle mice had higher *Tgfb1* (21‐fold, *p* < 0.0001) and *Acta2* (7.7‐fold, *p* < 0.0001) mRNA expression compared to young plasma. Compared to old vehicle plasma, exposure to old fisetin plasma reduced *Tgfb1* (−88%, *p* < 0.0001) and *Acta2* (−89%, *p* < 0.0001) mRNA expression (Figure 6D,E, Figure S6C,D). The addition of LIT‐927 to old vehicle plasma reduced *Tgfb1* (−83%, *p* < 0.0001) and *Acta2* (−76%, p < 0.0001) mRNA expression compared to old vehicle plasma alone (Figure 6D,E). The addition of CXCL12 to old fisetin plasma increased *Tgfb1* (5.4‐fold, *p* < 0.0001) and *Acta2* (5.4‐fold, p < 0.0001) mRNA expression compared to old fisetin plasma alone (Figure 6D,E, Figure S6C,D). In combination, these data indicate that the circulating SASP milieu and CXCL12 promote EndoMT in senescent ECs and that these age‐related changes in cell phenotypes are prevented by fisetin supplementation.

## 4 Discussion

In the present study, we assessed vascular cellular senescence at single‐cell resolution in the context of aging and senolytic treatment with fisetin. Our findings demonstrate that ECs are particularly susceptible to undergoing cellular senescence with aging and that fisetin, a naturally occurring senolytic compound, effectively eliminates senescent ECs in old animals. Across senescent ECs and in circulation, several SASP‐associated factors increased with aging and were reduced by fisetin supplementation. Among these, *Cxcl12* mRNA showed one of the most pronounced changes in association with aging and fisetin treatment; however, given the small size of the endothelial subpopulation and the systemic effects of fisetin, we cannot attribute circulating CXCL12 levels entirely to this cluster, though ECs appear capable of amplifying this response. Regardless of the tissue of origin, our findings demonstrate that the circulating SASP milieu contributed to age‐related endothelial dysfunction and the effects of the circulating SASP milieu were largely mediated by CXCL12. Fisetin‐induced reductions in circulating CXCL12 contribute to the beneficial effects of fisetin on endothelial function by mitigating mitochondrial oxidative stress, improving NO production, and favorably modulating endothelial transdifferentiation. These results provide novel insights into the role of senescent cells and the SASP in vascular aging and the potential of senolytic therapy to mitigate age‐related endothelial dysfunction.

Vascular endothelial dysfunction is a key risk factor, indicator, and predictor for CVD. Cellular senescence has emerged as a potent underlying mechanism of age‐related endothelial dysfunction and a likely contributor to CVDs, including atherosclerosis (Honda et al. 2021), hypertension (Westhoff et al. 2008), and occlusive stroke (Torres‐Querol et al. 2021). Previous studies have characterized age‐related increases in cellular senescence burden in aortic samples from mice (Xie et al. 2022), primates (Zhang et al. 2020), and humans (Li et al. 2020). Excess senescent cells accumulate in the vasculature among various mammals to promote chronic, low‐grade inflammation and excess oxidative stress through the SASP (Hu et al. 2022; Suda et al. 2023). But despite this initial evidence of vascular health improvements with senolytic treatment, there is a lack of information on EC senescence in vivo and the effects of senolytic treatment on ECs. In the present study, aortic senescent cell characterization identified ECs to develop a high cellular senescence burden with aging, which was reduced by senolytic treatment with fisetin. Previous studies have demonstrated that fisetin reduces whole‐aorta cell senescence burden in vivo (Mahoney, Venkatasubramanian, et al. 2023) as well as in cultured senescent ECs in vitro (Mahoney, Venkatasubramanian, et al. 2023; Zhu et al. 2017). At a single‐cell level, our study explored the effects of cellular senescence burden with aging and following in vivo fisetin supplementation across multiple cell types and identified that ECs undergo cellular senescence with aging and can be eliminated by in vivo senolytic treatment with fisetin. Importantly, the senescent EC population that we identified was involved in pathways related to CV pathologies and revealed a high expression of SASP‐related transcripts. As such, our findings align with previous studies reporting the efficacy of senolytic agents in reducing senescent cell burden in aging tissues. However, this study provides the first evidence of the in vivo effects of senolytic treatment on vascular cell senescence at a single‐cell level to reveal underlying mechanisms of age‐related endothelial dysfunction.

The circulating SASP milieu is composed of a heterogeneous collection of pro‐oxidant and pro‐inflammatory factors that vary depending on cell origin and cellular senescence induction. As such, the circulating SASP milieu is considered a major mediator of the adverse effects of senescent cells, but direct evidence for the impact of the SASP on endothelial function remains minimal. Emerging evidence indicates the circulating SASP milieu as a driver of age‐related endothelial dysfunction (Mahoney et al. 2024); however, the specific component(s) of the circulation responsible for transducing impairments in endothelial function remain to be elucidated. One of the key findings in the present study is the elucidation of the role of the circulating SASP milieu as an underlying mechanism of age‐related endothelial dysfunction. Our plasma exposure experiments in isolated arteries establish a causal role for the aged circulating SASP milieu in impairing endothelial function, with these age‐related impairments being largely prevented in old animals that received senolytic treatment with fisetin.

We next sought to determine candidate factors in the circulating SASP milieu that may be responsible for mediating endothelial dysfunction with aging, as identification of these molecules would provide insight into mechanisms by which cellular senescence drives endothelial dysfunction and establish novel therapeutic targets. The chemokine encoded by *Cxcl12* mRNA emerged as a factor most highly upregulated in senescent ECs with aging, with its expression reduced following fisetin senolytic treatment as determined by single‐cell transcriptomics. Additionally, the concentration of CXCL12 was elevated in the plasma of old animals and restored to young levels by fisetin supplementation. The addition and inhibition of CXCL12 in our plasma exposure experiments support a causal role for CXCL12 in age‐related endothelial dysfunction, as inhibition of CXCL12 in old vehicle plasma improved endothelial responses and adding exogenous CXCL12 to young plasma induced endothelial dysfunction. Moreover, the addition of CXCL12 to old fisetin plasma recapitulated age‐related endothelial dysfunction, indicating that senolytic treatment with fisetin mitigated circulating SASP milieu‐related endothelial dysfunction, in part, by targeting CXCL12. Collectively, these findings establish an altered circulating SASP milieu as an underlying mechanism by which senolytic treatment with fisetin improves endothelial function with aging.

The circulating SASP milieu stimulates endothelial dysfunction through underlying mechanisms that promote chronic inflammation and oxidative stress (Bloom et al. 2023; Mahoney et al. 2025). Notably, the pro‐inflammatory circulating SASP milieu may promote cellular senescence systemically and in nearby cells, further promoting tissue‐level dysfunction (Coppé et al. 2010). Senescent ECs also contain dysfunctional mitochondria which are key regulators of the SASP (Wiley et al. 2016). Dysfunctional mitochondria in senescent ECs produce excess ROS, which scavenges NO to reduce its bioavailability (Hayashi et al. 2008). Here, we showed that the exposure of young, isolated mouse arteries and cultured arterial ECs to the aged circulating SASP milieu induced humoral cellular senescence, reduced NO production, and stimulated mitochondrial oxidative stress. Importantly, these age‐related manifestations were prevented in arteries and ECs exposed to plasma from old animals supplemented with fisetin. Importantly, this approach allowed us to model the integration of both systemic and locally secreted factors and suggests that fisetin favorably modulates the circulating SASP milieu to prevent vascular aging mechanisms that drive endothelial dysfunction.

Given that CXCL12 has been implicated in inflammatory (Döring et al. 2019; Lu et al. 2024) and cellular senescence‐related pathways (Saul et al. 2022; Guduric‐Fuchs et al. 2024), its effects as a circulating SASP factor provide mechanistic insight into how endothelial dysfunction develops with aging. CXCL12 regulates the transduction of downstream signaling pathways involved in the EndoMT, a process whereby ECs lose their inherent endothelial phenotypes and gain enhanced contractile properties (Ara et al. 2005; Döring et al. 2019; Lu et al. 2024). The circulating SASP milieu‐mediated endothelial dysfunction was largely stimulated by independent effects of CXCL12 via humoral cellular senescence induction, reduced NO production, and stimulated mitochondrial oxidative stress. While our data demonstrate that CXCL12 is a potent driver of endothelial dysfunction, the exact directionality—whether primarily driven by systemic levels or local autocrine/paracrine signaling within the vessel wall—remains to be fully elucidated. Previous studies demonstrate that EC‐derived CXCL12 promotes atherosclerosis progression (Döring et al. 2019) and our findings support that CXCL12 induces endothelial dysfunction likely through the EndoMT, a manifestation that is prevented by senolytic treatment. As such, it is likely that the observed dysfunction results from an integrated signaling environment where ECs function as both a source and a target of CXCL12. The ability of fisetin to ameliorate CXCL12‐driven endothelial dysfunction further underscores its therapeutic potential in mitigating vascular aging.

## 5 Limitations

One limitation of the current study is that while we demonstrated that fisetin supplementation reduces circulating CXCL12, the systemic tissue distribution of fisetin and CXCL12 must be considered. Fisetin is known to distribute widely into tissues including the liver, kidneys, and brain, after oral administration (Yousefzadeh et al. 2018). As such, it is likely that fisetin works pleiotropically across multiple tissues to reduce the systemic production of CXCL12. Furthermore, while we identified *Cxcl12* as a key SASP factor within a specific EC cluster, we cannot definitively conclude that senescent ECs are the sole source of systemic CXCL12. Moreover, the current study design does not distinguish between the effects of circulating CXCL12 and potential autocrine/paracrine signaling within the endothelium. While senescent endothelial cluster 10 represents a transcriptomic source, the relative contribution of local versus systemic CXCL12 to the observed phenotype requires further investigation using cell‐specific knockout models. Finally, while the CXCL12/CXCR4/ACKR3 axis is a well‐known regulator of vascular homeostasis (Wiley et al. 2016), our study did not employ receptor inhibition to confirm if the observed level of cellular senescence is a direct result of CXCL12 signaling or a secondary effect of the broader circulating SASP milieu. Future studies utilizing CXCL12 administration and receptor inhibition are necessary to determine whether CXCL12‐induced senescence is a direct receptor‐mediated event or a consequence of the broader systemic environment.

## 6 Conclusions

In conclusion, this study identifies that senescent ECs accumulate with advancing age and promote the secretion of SASP factors into circulation. Senolytic intervention with fisetin effectively eliminated senescent ECs and highlighted CXCL12 as a critical SASP factor that is increased with advanced age and reduced by senolytic treatment. Mechanistically, we uncovered that the circulating SASP milieu and CXCL12 induce cellular senescence, reduce NO production, promote mitochondrial oxidative stress, and stimulate EndoMT to confer endothelial dysfunction. By demonstrating that senolytic treatment with fisetin effectively reduces CXCL12 levels and improves endothelial function, our findings provide mechanistic insight into the therapeutic potential of senolytic compounds in vascular aging. Importantly, these findings may have important implications for ongoing clinical trials investigating the efficacy of fisetin supplementation for improving endothelial function in healthy mid‐life and older adults (NCT06133634). These results provide physiological and mechanistic insight that supports the development, establishment, and translation of senolytic therapies to improve cardiovascular health with aging.

## Author Contributions

Sophia A. Mahoney, Krystyna Mazan‐Mamczarz, Dimitrios Tsitsipatis, Myriam Gorospe, Douglas R. Seals, Matthew J. Rossman, Allison B. Herman, and Zachary S. Clayton designed the study. Sophia A. Mahoney, Krystyna Mazan‐Mamczarz, Dimitrios Tsitsipatis, Nicholas S. VanDongen, CH, Ada N. Okereke, Rachel Munk, Sanna Darvish, Kevin O. Murray, SD, and Zachary S. Clayton performed the experiments. Sophia A. Mahoney, Krystyna Mazan‐Mamczarz, Dimitrios Tsitsipatis, Rachel Munk, Sanna Darvish, Allison B. Herman, and Zachary S. Clayton analyzed the data. Sophia A. Mahoney, Krystyna Mazan‐Mamczarz, Dimitrios Tsitsipatis, Sanna Darvish, Kevin O. Murray, Myriam Gorospe, Douglas R. Seals, Matthew J. Rossman, Allison B. Herman, and Zachary S. Clayton interpreted the data and wrote the manuscript. All authors read and approved the final version of the manuscript.

## Funding

This research was supported in part by the Intramural Research Program of the National Institute on Aging. This work was supported by National Institute of Health Grants F31 HL165885 (to Sophia A. Mahoney), F31 AG087709 (Supriyo De), F32 HL167552 (Kevin O. Murray), K01 DK115524 (to Matthew J. Rossman), K99 HL159241 (to Zachary S. Clayton), R21 AG078408 (to Douglas R. Seals and Zachary S. Clayton), R01 AG055822 (to Douglas R. Seals), and R01 AG055822‐S1 (to Douglas R. Seals and Zachary S. Clayton) and an American Heart Association award AHA 23CDA1056582 (to Matthew J. Rossman).

## Conflicts of Interest

The authors declare no conflicts of interest.

## Supporting information


**Data S1:** acel70500‐sup‐0001‐DataS1.docx.

## Data Availability

RNA sequencing data are deposited in GSE239591 (token: sfqjscwkrrezpyh) and GSE239602 (token: itypuqwkhhwtlit).
